# Stress-Induced Sulfide Production by *Bacillus subtilis* and *Bacillus megaterium*

**DOI:** 10.3390/microorganisms12091856

**Published:** 2024-09-07

**Authors:** Alexey Tyulenev, Galina Smirnova, Vadim Ushakov, Tatyana Kalashnikova, Lyubov Sutormina, Oleg Oktyabrsky

**Affiliations:** Institute of Ecology and Genetics of Microorganisms, Perm Federal Research Center, Russian Academy of Sciences, Goleva 13, Perm 614081, Russia; leksey333@yandex.ru (A.T.); smirnova@iegm.ru (G.S.); ushakovvad@yandex.ru (V.U.); tatyana-kalashnikova22@yandex.ru (T.K.); lyubov-sutormina@mail.ru (L.S.)

**Keywords:** sulfide production, stresses, *Bacillus subtilis*, *Bacillus megaterium*, electrochemical sensors

## Abstract

It was previously discovered that, in the Gram-negative bacterium *Escherichia coli* growing on a minimal medium with sulfate, stress-induced growth arrest is accompanied by the release of hydrogen sulfide. The source of the sulfide is the desulfurization of intracellular cysteine as one of the ways of maintaining it at a safe level. The danger of excess cysteine is associated with its participation in the Fenton reaction, leading to the formation of highly toxic hydroxyl radicals. Using electrochemical sensors, we identified stress-induced sulfide production in the Gram-positive bacteria *Bacillus subtilis* and *Bacillus megaterium*, growing on a minimal medium with sulfate, and changes in physiological parameters such as Eh, pH, and oxygen and potassium consumption. Sulfide production was observed during growth arrest due to the depletion of glucose, ammonium or antibiotic action. The use of sensors allowed to continuously record, in growing cultures, even small changes in parameters. There were significant differences in the amount and kinetics of sulfide production between *Bacillus* and *E. coli*. These differences are thought to be due to the lack of glutathione in *Bacillus*. It is suggested that stress-induced sulfide production by *Bacillus* under the described conditions may be one of the previously unknown sources of hydrogen sulfide in nature.

## 1. Introduction

A sharp drop in the redox potential (Eh jump), measured using a platinum electrode, was previously discovered during stress-induced growth arrest of the Gram-negative bacterium *Escherichia coli*, growing on a minimal medium with sulfate as the sole sulfur source. A close correlation was found between the stress-induced changes in Eh and the level of low-molecular-weight thiols (LWTs) in the medium [1]. Our further studies showed that, in *E. coli*, stress-induced Eh jumps are the result of hydrogen sulfide (H_2_S) excretion. Sulfide production is induced during growth arrest caused by glucose depletion, isoleucine starvation, or antibiotic exposure [2,3]. The production of sulfide has been observed in *E. coli* growing on M9 medium containing thiosulfate as the sole inorganic sulfur source in response to glucose starvation [4].

In *E. coli*, the formation of sulfide by cysteine desulfurization may be one of the ways of maintaining cysteine homeostasis under stress-induced conditions [2,3]. L-cysteine is used for protein and glutathione synthesis and as a source of reduced sulfur in other organic molecules. However, even at subtoxic concentrations, cysteine can inhibit several enzymes of amino acid synthesis and impair bacterial growth [5]. Cysteine can also promote the Fenton reaction in the presence of H_2_O_2_ to form toxic hydroxyl radicals that cause oxidative damage to critical biomolecules, including the DNA [6]. Therefore, the intracellular level of cysteine is strictly regulated by various pathways, including biosynthesis, transport, and degradation [7].

In mammals, H_2_S is an important endogenous gasotransmitter and signaling molecule [8]. There are increasing data on the important role of endogenously produced H_2_S in bacterial physiology. In cultures growing on cysteine-containing media, H_2_S can protect bacteria from antibiotic-induced damage [9] and oxidative stress [10], and it is involved in a bacterial defense mechanism against the host immune response [11]. Modulation of endogenous hydrogen sulfide levels can significantly alter the sensitivity of bacteria, including pathogens, to antibiotics [12].

In aerobic cultures of Gram-positive bacteria *Bacillus subtilis* and *Bacillus megaterium* growing on a minimal medium, characteristic Eh jumps to negative values upon growth cessation caused by glucose and ammonium exhaustion have also been observed. As with *E. coli*, a correlation between the stress-induced changes in Eh and the level of low-molecular-weight thiols in the medium was found [1].

*Bacillus* are widespread in nature and play an active part in the decomposition of organic matter. The ecological niches of *B. subtilis* and *B. megaterium* are located in soil, and they do not produce sulfide during normal growth on sulfate as a sulfur source. It was of interest to test whether *Bacillus* produces sulfide under stressful conditions. In this work, using five electrochemical sensors, for the first time, we identified stress-induced sulfide production in *B. subtilis* and *B. megaterium*, as well as changes in physiologically important parameters, such as Eh, pH, and oxygen and potassium consumption, which accompany sulfide leakage. There were significant differences in the amount and kinetics of sulfide production between *Bacillus* and *E. coli*. These differences are thought to be due to the lack of the tripeptide glutathione in *Bacillus*.

## 2. Materials and Methods

### 2.1. Bacterial Strains and Growth Conditions

The strains of *Bacillus subtilis* VKM428 and *Bacillus megaterium* VKM512 from the Russian collection of microorganisms (VKM) and *Escherichia coli* BW25113 from the Keio collection (*E. coli* Genetic Stock Center) were used in this study. Overnight cultures were grown aerobically at 37 °C in 250 mL flasks with shaking at 150 rpm in M9 minimal medium [13] supplemented with glucose (5 g L^−1^) as the sole carbon and energy sources. In the experiments with glucose starvation, after centrifugation, the cells were transferred to 100 mL of M9 medium containing 0.25 g L^−1^ glucose to a final optical density at 600 nm (OD_600_) of 0.1 and cultured as indicated above. For the experiments with nitrogen starvation, the amount of NH_4_Cl in the M9 medium was reduced to 0.05 g L^−1^, and the amount of glucose was 10 g L^−1^. Substrate exhaustion and the onset of starvation were confirmed by the cessation of growth by measuring OD_600_. The antibiotics chloramphenicol (25 µg mL^−1^), kanamycin, tetracycline, and erythromycin (30–120 µg mL^−1^) were added to the culture growing on M9 medium with 10 g L^−1^ glucose when the OD_600_ reached 0.4.

### 2.2. Real-Time Monitoring of Eh, Dissolved Oxygen (dO_2_), pH, and Ions of Extracellular Sulfide (S^2−^) and Potassium (K^+^)

This work used highly sensitive electrochemical sensors immersed directly in the culture medium, which made it possible to record the measured parameters continuously and simultaneously and detect even small changes, which is difficult when using other methods. The use of electrochemical sensors is described in more detail in our previous works [2,3]. Four electrochemical sensors were used to monitor the physiological state of the bacterial cultures. The redox potential (Eh) in the bacterial cultures was measured using platinum and reference electrodes and Mettler Toledo SevenCompact™ pH/Ionmeters S220 (Mettler Toledo, Greifensee, Switzerland). The dissolved oxygen (dO_2_) and pH were measured using a Clarke oxygen electrode InPro 6800 (Mettler Toledo, Greifensee, Switzerland) and a combined pH electrode InLab Expert ProISM (Mettler Toledo, Greifensee, Switzerland), respectively. The dO_2_/pH controller of a BioFlo 110 fermentor (New Brunswick Scientific Co., Edison, NJ, USA) was used for data recording. Changes in the levels of extracellular K^+^ were registered using the system of K^+^-selective (ELIS121K) (IT Company, Moscow, Russia) and reference electrodes and a computer pH/ion meter cpX-2 (IBI, Pushchino, Russia). For the K^+^ measurements, cells were grown as described above, except for the medium, which contained a low K^+^ concentration (0.2 mM).

The extracellular sulfide levels were detected using the system of sulfide-specific ion-selective electrodes XC-S^2—^001 (Sensor Systems Company, St. Petersburg, Russia), a reference electrode, and a computer pH/ion meter cpX-2 (IBI, Pushchino, Russia). The electrode had a sensitivity threshold of 10 nM, operated in a wide pH range (6 ÷ 12), and did not respond to changes in oxygen in the cultivation environment. The sulfide concentration in the medium was calculated using a standard curve prepared with known amounts of Na_2_S. For the calculations, the value of the difference between the maximum drop in the potential of the sulfide electrode after exposure to the studied factor and its value in the absence of any effects was used. The synchronous processing of all primary data from the sensor system was carried out using the RS-232 and Modbus protocols and the Advantech OPC Server v3.0 software package.

The H_2_S levels in the gas phase were estimated using lead acetate [Pb(Ac)_2_], which reacts specifically with H_2_S to form a brown lead sulfide stain [10]. In our conditions, this method had a sensitivity of 0.1 μM. Lead acetate-soaked paper strips were affixed in culture flasks above the level of the liquid culture. To determine the total H_2_S, the paper strip was left for the entire duration of the experiment. The spots were scanned and then quantified using ImageJ1.54g. The results were expressed as arbitrary units.

### 2.3. Determination of Intracellular and Extracellular Cysteine

For the intracellular L-cysteine assays, 40 mL of culture (20 mL each from two identical flasks) was centrifuged (8000× *g* for 5 min), suspended in 4 mL of 0.1 M Tris-HCl pH 8.6, and lysed by sonification at 0 °C, using a 30 s pulse for six cycles. Perchloric acid (final concentration of 0.5 M) was added to the lysate to precipitate proteins. After 30 min, the suspension was centrifuged (8000× *g* for 5 min), the supernatant was adjusted to a pH of 8.6 with KOH, frozen, centrifuged to eliminate the potassium perchlorate, and evaporated using a rotary evaporator RV10 (IKA, Staufen, Germany) at 65 °C to 0.57 mL and then treated with 0.25 mL of dithiothreitol (10 mM) for 10 min. Reduced samples (0.5 mL) were used to determine the amount of cysteine according to the Gaitonde method, as described previously [14]. Standard curves were generated with known amounts of cysteine, which were treated as samples of cell suspensions.

### 2.4. Determination of Total Catalase Activity

The total catalase activity was measured by a spectrophotometric method [15]. To prepare the extracts, *E. coli* samples (20 mL) were centrifuged (5 min, 8000× *g*) and resuspended in 4 mL of 5 mM potassium phosphate buffer (pH 7.0), containing 5 mM EDTA, 10% glycerol, and 25 μmol phenylmethylsulfonyl fluoride, and then disrupted by sonication on ice (four rounds of five 5 s pulses). The soluble cell fraction was separated by centrifugation (10 min at 12,000× *g*) at 4 °C. The total protein concentration in the supernatant was measured by the method of Lowry. The specific catalase activity was expressed in micromoles of H_2_O_2_ decomposed per minute per milligram of total protein.

### 2.5. Statistical Analysis of the Data

Each result is indicated as the mean value of three-to-five independent experiments ± the standard error of the mean (SEM). Significant differences were analyzed by Student’s *t*-test. A *p*-value of 0.05 was used as the cut-off for statistical significance. The results were analyzed by means of the program packet Statistica 8.0.360 (StatSoft Inc., Tulsa, OK, USA, accessed on 27 August 2007).

## 3. Results

### 3.1. Glucose and Ammonium Depletion in B. subtilis and E. coli

It has previously been shown that, in *E. coli* and *B. subtilis* growing on aerobic media with sulfate as a sulfur source, the transition from exponential growth to glucose starvation is accompanied by a rapid drop in the potential of the platinum electrode measuring Eh. In *E. coli*, this Eh jump has later been shown to be associated with sulfide excretion [2]. In our study, it was of interest to check whether there was a relationship between the changes in Eh and extracellular sulfide in this situation in *B. subtilis*.

**Figure 1:**
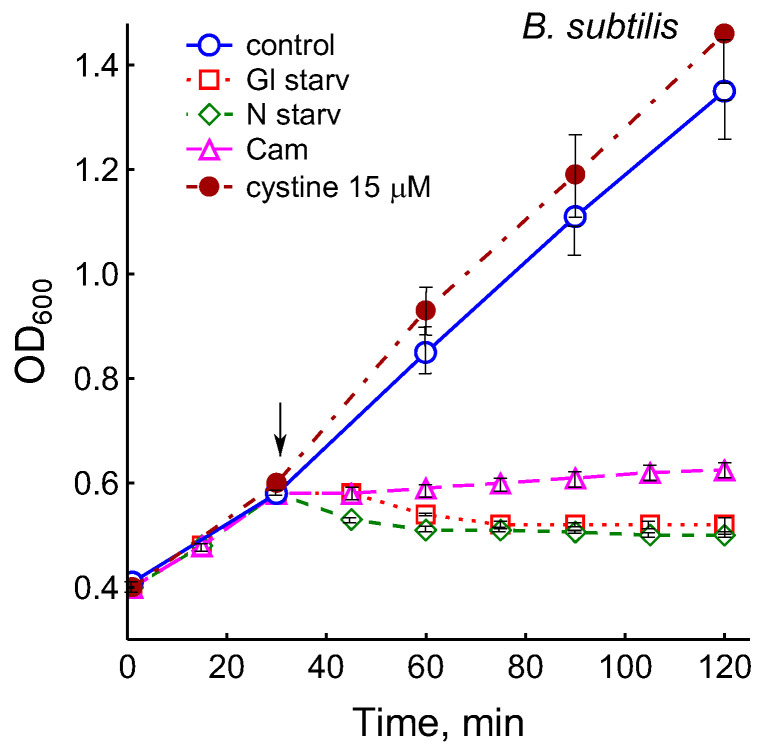
Treatment with 25 µg mL^−1^ chloramphenicol (CAM) and glucose or ammonium depletion (but not cystine addition) causes *B. subtilis* growth arrest.

**Figure 2:**
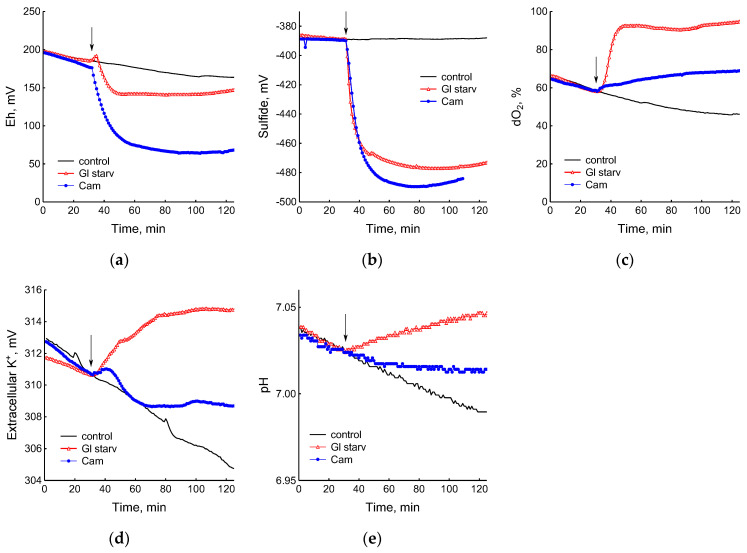
Changes in platinum (**a**) or sulfide-selective (**b**) electrode potential, dissolved oxygen (dO_2_) level (**c**), potassium-selective electrode potential (**d**), and pH (**e**) in *B. subtilis* in response to glucose depletion or the addition of chloramphenicol (25 µg mL^−1^). The arrows indicate the time of CAM addition or the onset of glucose starvation.

**Figure 3:**
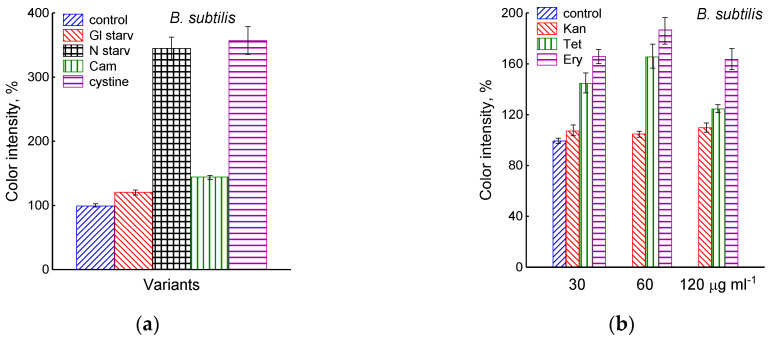

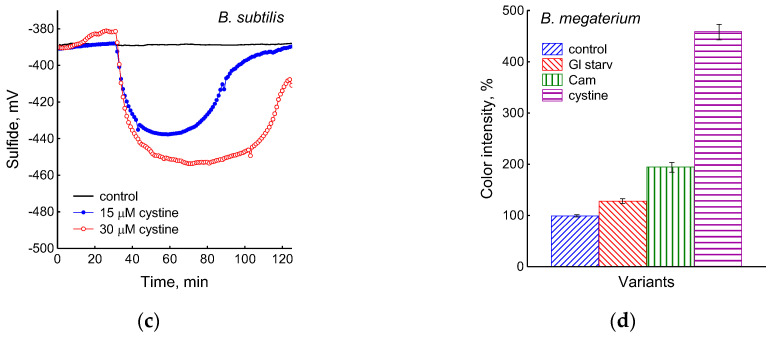
Extracellular H_2_S accumulation in *B. subtilis* was determined using Pb(Ac)_2_ strips after incubation for 120 min under the tested conditions. Ammonium or glucose starvation and CAM or cystine supplementation (**a**), treatment with various concentrations of kanamycin, tetracycline, or erythromycin (**b**). Changes in sulfide electrode potential upon treatment of *B. subtilis* with cystine (**c**). Extracellular H_2_S accumulation in *B. megaterium* was determined using Pb(Ac)_2_ after the addition of chloramphenicol (25 µg mL^−1^) or cystine (15 µM) and glucose depletion (**d**).

The growth arrest of *B. subtilis* due to glucose depletion (Figure 1) was accompanied by a rapid irreversible drop in the potential of the platinum and sulfide sensors, indicating an increase in extracellular sulfide (Figure 2a,b). The largest deviation in the potential of the sulfide sensor from the baseline was 73 ± 6 mV, which corresponded to 510 nM sulfide. The test using Pb(Ac)_2_ strips showed an increase in sulfide production during glucose starvation by 21 ± 4 conventional units (Figure 3a and Figure S1).

The comparison of the profiles of changes in the potentials of the platinum and S^2−^-selective electrodes (Figure 2a,b) indicates that, as in *E. coli* [2], changes in Eh during glucose starvation in *B. subtilis* are also associated with sulfide leakage. At the same time, unlike *E. coli*, in *B. subtilis* the excretion of sulfide upon glucose depletion is irreversible. In both bacteria, under normal growth conditions (control) extracellular sulfide is not detected by either of the methods used.

Data obtained using electrochemical sensors show that the growth arrest of *B. subtilis* caused by the exhaustion of glucose was accompanied by a decrease in metabolic activity, as evidenced by a rapid and almost complete decrease in oxygen consumption and the release of potassium from the cells (Figure 2c,d). Acidification of the medium was replaced by alkalization (Figure 2e), which may be due to the consumption of organic acids accumulated during glucose catabolism.

In response to glucose starvation, the cysteine levels in *B. subtilis* increased by 33% in the cells and 30% in the medium (Figure S2). Notably, glucose depletion in *E. coli* growing under the same conditions was not accompanied by extracellular cysteine accumulation [2].

It has previously been shown that, as in the case of glucose, the growth arrest of *B. subtilis* and *E. coli* under ammonium depletion is accompanied by a rapid drop in Eh [1]. In this study, it was of interest to test whether *B. subtilis* produced sulfide during ammonium starvation and simultaneously compare the responses of *B. subtilis* and *E. coli* to this stress. Whether *E. coli* produced sulfide during ammonium starvation was also unknown.

**Figure 4:**
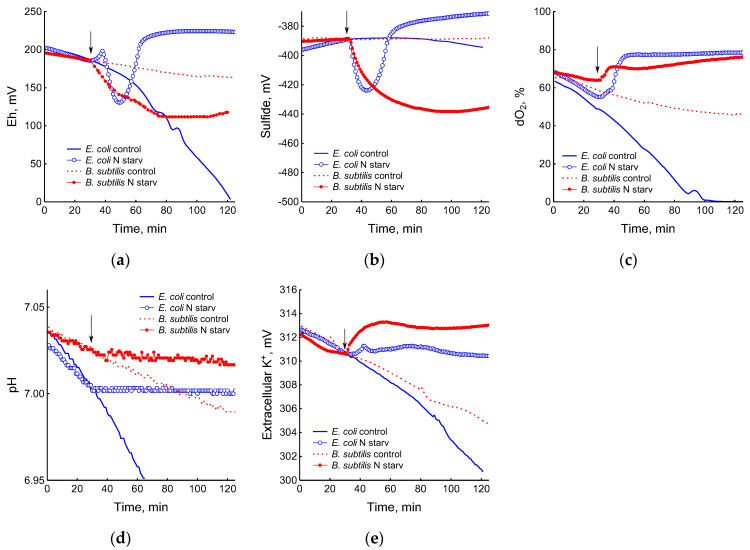
Changes in platinum (**a**) or sulfide-selective (**b**) electrode potential, dissolved oxygen (dO_2_) level (**c**), pH (**d**), and potassium-selective electrode potential (**e**) in the response of *B. subtilis* and *E. coli* to ammonium depletion. The arrows indicate the time of the onset of ammonium starvation.

The depletion of ammonium in the medium led to a rapid and complete cessation of growth of *B. subtilis* and *E. coli* (Figure 1). In response to growth arrest, a drop in the potential of the platinum and sulfide electrodes was observed in both *B. subtilis* and *E. coli* (Figure 4a,b). The high agreement between the time profiles of changes in Eh and the sulfide sensor for each of the two bacteria indicates the contribution of sulfide to the change in Eh in response to stress. The largest deviation of the sulfide electrode potential from the baseline was 48 ± 3 mV in *B. subtilis* and 35 ± 2 mV in *E. coli* (corresponding to 260 and 80 nM sulfide). Thus, under ammonium depletion, sulfide production in *B. subtilis* was more than three times higher than in *E. coli.* As in the case of glucose [2], the leakage of sulfide under ammonium depletion in *E. coli* is reversible and includes a phase of rapid leakage lasting about 10 min and a phase of slower return to baseline values. In *B. subtilis*, sulfide leakage is irreversible and proceeds much slower than in *E. coli* (Figure 4a,b). An independent test using Pb(Ac)_2_ strips showed a simultaneous increase in H_2_S over the culture medium in *B. subtilis* (Figure 3a and Figure S1).

The growth arrest of *B. subtilis* and *E. coli* caused by the depletion of ammonium was accompanied by a decrease in oxygen consumption (Figure 4c) and the cessation of medium acidification (Figure 4d). As in the case of glucose, in response to ammonium starvation, *B. subtilis* lost some of the intracellular potassium. It is remarkable that *E. coli* cells retained potassium at a level close to that which was in the growing cells up until the onset of ammonium starvation (Figure 4e).

Importantly, in *B. subtilis*, the addition of glucose or ammonium during the starvation response quickly restores growth and metabolic activity, as evidenced by renewed oxygen consumption and a decrease in pH. An increase in the potential of the sulfide sensor indicated a decrease in the sulfide content in the medium, which may have been due to its involvement in the sulfur metabolism via growing cells (Figure S3).

### 3.2. Exposure of B. subtilis to Antibiotics

The treatment of growing *B. subtilis* with 25 μg mL^−1^ chloramphenicol caused a rapid cessation of growth (Figure 1) and an irreversible decrease in the potential of Eh and sulfide sensors (Figure 2a,b). The largest deviation from the baseline was 108 ± 3 mV, which corresponded to 1.35 µM sulfide (Figure 2b). The Pb(Ac)_2_ strip test also showed an increase in the H_2_S content over the culture medium after the addition of Cam. Besides chloramphenicol, H_2_S excretion in *B. subtilis* was induced by erythromycin, kanamycin, and tetracycline (Figure 3a,b and Figure S3). H_2_S excretion was previously observed by us when *E. coli*, growing on M9 medium with glucose, was treated with several antibiotics, including chloramphenicol (Cam) and tetracycline (Tet) [2].

The use of electrochemical sensors made it possible to identify significant differences in the response of *B. subtilis* to glucose starvation and the effect of chloramphenicol. Although in both cases there was a complete cessation of growth, the cells treated with the antibiotic showed noticeable metabolic activity, continuing to consume glucose and oxygen and retain potassium, but at a lower intensity than the untreated cells (Figure 2c–e).

The exposure of *B. subtilis* to chloramphenicol did not cause significant changes in free intracellular cysteine, but stimulated the accumulation of extracellular cysteine to 0.95 µM/OD_600_, more than twice its level in the control (Figure S2). Under similar conditions, *E. coli* responded to treatment with chloramphenicol in the same way, accumulating 1.05 µM/OD_600_ cysteine in the medium [3]. It should be noted that M9 medium containing 10 g L^−1^ glucose was used when studying the effect of chloramphenicol on the level of extra- and intracellular cysteine in growing *B. subtilis*. This prevented growth arrest due to exhaustion of the carbon and energy source. In experiments with glucose starvation, M9 medium containing 0.25 g L^−1^ glucose was used (Section 2.1) This may explain why, in the latter case, the basal level of extracellular cysteine was significantly lower than in the medium with a high glucose content. The absence of glucose could also have been the reason for the slower accumulation of extracellular cysteine during starvation than after chloramphenicol treatment (Figure S2). Like sulfide, cysteine has a high redox activity, and an increase in its extracellular concentration may contribute to a decrease in the redox potential of the platinum electrode during glucose starvation and chloramphenicol treatment.

Remarkably, the continuous and simultaneous recording of changes in the sulfide electrode potential and other parameters (Eh, dO_2_, pH, K^+^) indicated that the cells quickly responded to all the stresses tested. The release of sulfide from the cells was recorded a few seconds after exposure to a stress-inducing factor and occurred synchronously with changes in other parameters.

### 3.3. Response of B. subtilis to the Addition of Cystine

It was previously reported that the addition of cysteine or cystine to *B. subtilis* and *E. coli* growing on a minimal medium with sulfate leads to the release of hydrogen sulfide [16]. In another study, cystine treatment of *E. coli* growing on a medium with glycerol and sulfate also led to the release of sulfides [17]. In their work, hydrogen sulfide was determined using the methylene blue method.

It was of interest in this study to test this effect in *B. subtilis* under our conditions and investigate the action of exogenous cystine on the growth and other physiological parameters in these bacteria. Monitoring changes in the potential of the sulfide-selective electrode showed that the addition of cystine to *B. subtilis* growing on M9 medium with sulfate resulted in reversible sulfide release. The amplitude changes in pS^2−^ upon the addition of 15 or 30 μM cystine were 50 ± 14 (295 nM) and 103 ± 11 mV (1.3 μM), respectively (Figure 3c). The Pb(Ac)_2_ strip test confirmed H_2_S excretion after the addition of cystine to growing *B. subtilis* (Figure 3a). The addition of cystine at the indicated concentrations did not stop the growth (Figure 1) and did not affect the rate of oxygen consumption, in line with the data reported earlier for *E. coli* [17]. There was also no effect of cystine on parameters such as the pH and the content of potassium ions.

### 3.4. Catalase Activity in B. subtilis and E. coli

In the mid-exponential growth phase, the catalase activity of *B. subtilis* growing under aerobic conditions on M9 medium was 5.2 times higher than in *E. coli* under the same conditions, amounting to 107 ± 13 and 20.7 ± 2.3 μmol H_2_O_2_/min × mg protein, respectively.

### 3.5. H_2_S Excretion in B. megaterium

Using the Pb(Ac)_2_ strip test, it was found that the stress-induced excretion of H_2_S was also detected in another Gram-positive bacteria, *B. megaterium*. The amount of H_2_S excreted by *B. megaterium* under conditions of glucose exhaustion, treatment with 25 μg mL^−1^ chloramphenicol, or the addition of 30 μM cystine was equal to 28, 95, and 360 arbitrary units, respectively (Figure 3d and Figure S4). Compared with *B. subtilis*, *B. megaterium* cells excreted twice as much H_2_S under the treatment with chloramphenicol and almost the same under glucose exhaustion. No growth inhibition was observed with cystine.

## 4. Discussion

We previously reported that, in *E. coli* growing on minimal M9 medium with sulfate, stress-induced growth arrest is accompanied by sulfide production. Sulfide formation was a consequence of the desulfurization of intracellular cysteine, as one of the ways to reduce its excess, resulting from the inhibition of protein synthesis [2,3].

There are significant differences in the physiology of bacteria growing on media with cysteine or sulfate as the sulfur sources. The first case can occur in the life cycle of bacteria such as *E. coli*, for which the intestinal tract is the main ecological niche. For soil bacteria like *B. subtilis*, growth on sulfate media can happen quite often.

When *E. coli* grows in an aerobic environment containing cystine, cells import and reduce it to cysteine, which is then consumed for protein synthesis and other needs. To prevent the accumulation of cytoplasmic cysteine above dangerous levels, an inducible cysteine/cystine shuttle system is in place. Some part of cysteine is degraded by enzymes with desulfhydrase and desulfidase activity, followed by the release of sulfide [17,18,19]. In *E. coli* growing on a medium with sulfate as the sole source of sulfur, sulfate is transported into the cell, reduced to sulfide, and reacts with O-acetylserine to form cysteine [7]. In the present work, all studied bacteria were grown in minimal medium M9 with sulfate and did not produce sulfide during normal growth. Here, we showed that, as in *E. coli*, in aerobic *B. subtilis* grown on sulfate minimal medium, sulfide production is induced during growth arrest in response to glucose or ammonium depletion and antibiotic exposure.

In *B. subtilis*, the pathways involved in the synthesis of cysteine from sulfate and the subsequent metabolism are well characterized and largely similar to those found in *E. coli* [20,21,22,23]. As in other bacteria, in *B. subtilis*, cysteine desulfurization can be catalyzed by several enzymes [20] and be one of the sources of the stress-induced sulfide production observed here. At the same time, significant differences are observed in the kinetics of production and the amount of sulfide formed between *B. subtilis* and *E. coli*. Unlike *B. subtilis*, stress-induced sulfide release in *E. coli* is a short-term process, and, accordingly, the amount of sulfide accumulated in the medium is much less.

One possible explanation for the observed effect may be related to the use of different types of low-molecular-weight (LMW) thiols by these bacteria. *E. coli*, as other Gram-negative bacteria, contains tripeptide glutathione as the main LMW thiol and cysteine buffer, while in most Gram-positive bacteria it is absent [24]. In aerobic *E. coli*, glutathione is present in the millimolar range [25] and plays a number of important roles in the cellular metabolism [26]. When *E. coli* grows on a minimal medium with sulfate, the stress-induced excess of cysteine is mainly incorporated into glutathione (up to 90%), 7–9% of free cysteine is exported from the cells, and the share of hydrogen sulfide does not exceed 1–3% [3].

*B. subtilis,* as a functional analog of glutathione, can utilize bacillithiol (BSH) (α-anomeric glycoside of L-cysteinyl-D-glucosamine with L-malic acid) [27,28]. If, in growing *E. coli*, the intracellular concentration of GSH is two orders of magnitude higher than that of cysteine [3,29], then, in *B. subtilis*, the BSH concentration is close to that of cysteine, and the BSH content is about 30 times lower than GSH in bacteria producing this tripeptide [27]. Considering these data, BSH is not a very suitable buffer for cysteine. *B. megaterium*, as an LMW thiol, contains 0.4 mM coenzyme A (CoA), 0.1 mM BSH, and 0.17 mM cysteine and does not synthesize GSH [27]. CoA is considered an antioxidant cofactor, though not as a protected form of cysteine [30].

**Figure 5:**
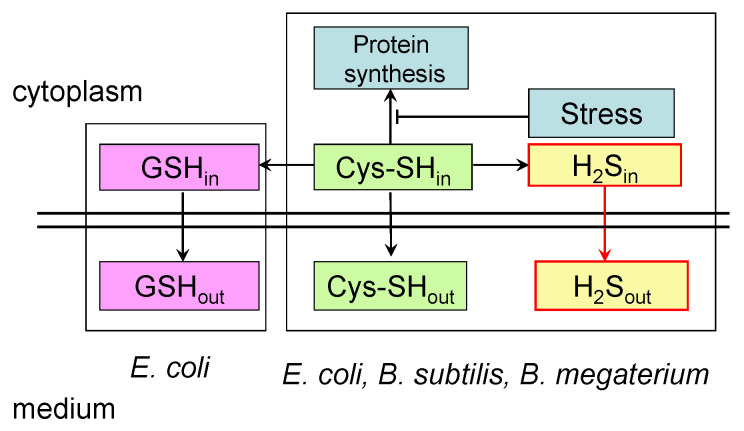
Proposed scheme of stress-induced excretion of low-molecular-weight thiols by *Bacillus* and *E. coli*. The stress-induced inhibition of protein synthesis leads to an increase in intracellular cysteine pools. In *B. subtilis*, *B. megaterium*, and *E. coli*, to restore cysteine homeostasis, one part of it is excreted into the medium, and the other part undergoes desulfurization with the formation of hydrogen sulfide, which diffuses into the medium. In *E. coli*, part of the cysteine is also incorporated into glutathione. It is assumed that this is the reason for the increased production of stress-induced sulfide in *Bacillus* compared to *E. coli*. GSH_in_ and GSH_out_ are intra- and extracellular glutathione pools; Cys-SH_in_ and Cys-SH_out_ are intra- and extracellular cysteine pools; H_2_S_in_ and H_2_S_out_ are intra- and extracellular hydrogen sulfide.

The data obtained in this study indicate that, in the absence of an LMW thiol similar to glutathione, *B. subtilis* cells resolve the problem of cysteine homeostasis by increasing desulfhydrase activity to a higher level than in *E. coli*. The response of *B. subtilis* to chloramphenicol suggests that cysteine export may make some contribution to maintaining its intracellular homeostasis, but, as in *E. coli*, this pathway is probably of minor importance. Interestingly, this mode of action is manifested in response to stress in *E. coli* mutants deficient in glutathione synthesis. In response to amino acid starvation and chloramphenicol treatment, these mutants increase H_2_S production, the level of intracellular cysteine, and the excretion of cysteine [3]. It appears that, under stress conditions, *Bacillus* behave, at least in part, like GSH-deficient *E. coli* mutants. A proposed scheme explaining the differences in the mechanisms of the stress-induced excretion of low-molecular-weight thiols in *Bacillus* and *E. coli* is shown in Figure 5.

In the course of this work, we identified another case in which the behavior of *B. subtilis* and mutants of *E. coli* lacking glutathione is phenotypically similar. The catalase activity of *B. subtilis* was found to be more than five times greater than that of wild-type *E. coli* under identical conditions (Section 3.4). We previously reported that total catalase activity and the expression of the *katG* and *katE* genes encoding HPI and HPII catalases were significantly higher in the GSH-deficient *E. coli* strain than in wild-type cells [26]. Thus, *B. subtilis* can compensate for the lack of glutathione in several ways.

Another factor that distinguishes *B. subtilis* from *E. coli* and may affect sulfide production is the ability of *B. subtilis* to sporulate. This factor can be ruled out, since, under our conditions, ammonium or glucose remained in the medium during starvation stress. It is known that the presence of these compounds in *B. subtilis* growing on minimal media prevents spore formation [31].

It is assumed that, in nature, hydrogen sulfide is generated mainly in the processes of dissimilatory sulfate reduction by sulfate-reducing bacteria. These microorganisms are ubiquitous in anoxic habitats and produce hydrogen sulfide during normal growth [32,33]. The ecological niches of *B. subtilis* and *B. megaterium* can be found in soil; they live primarily in an aerobic environment and do not produce sulfide during normal growth on sulfate as a sulfur source. The data obtained in this study, together with those we reported earlier for *E. coli*, indicate that the production of sulfide under the described conditions may be a universal reaction, induced under different types of stresses by different bacterial species, and one of the potential sources of sulfide in nature.

In the natural environment, *Bacillus* bacteria are constantly exposed to various stresses, including starvation and antibiotics. In these cases, conditions can be created for the production of sulfide in a wide range of concentrations. High levels of stress-induced sulfide can accumulate on the surface of bacterial biofilms growing on sulfate in oxygenated environments. The resulting sulfide may contribute to the adaptation of *Bacillus* to growth-inhibitory stresses and also play the role of a signal molecule in intercellular and inter-organismal communications.

*B. subtilis* and *B. megaterium* are plant-growth-promoting rhizobacteria (PGPR) that exhibit a significant interaction with plant roots and have positive effects on plant growth and the reduction in both biotic and abiotic stresses. These microorganisms enhance stress tolerance in their plant hosts by inducing the expression of stress-response genes, phytohormones, and stress-related metabolites [34]. PGPR have emerged as an alternative solution to the use of synthetic pesticides and fertilizers, which have toxic effects on human health and the environment [35,36]. In plants, the most important role of H_2_S is as an intermediate in sulfate assimilation. However, recently, evidence has accumulated on H_2_S functioning as a signaling molecule, participating in the regulation of plant stress response, particularly draught stress [37]. It can be assumed that the stress-induced production of H_2_S by *Bacillus* living in the rhizosphere can have a positive effect on plant growth, increasing plants’ resistance to adverse factors. The data obtained in this study may contribute to the understanding of the mechanisms of interaction between rhizosphere *Bacillus* and plants.

## 5. Conclusions

Using five electrochemical sensors, we identified stress-induced sulfide production in the Gram-positive bacterium *Bacillus subtilis*, growing on a minimal medium with sulfate, as well as changes in the physiological parameters that accompany sulfide leakage. The use of sensors made it possible for us to continuously and synchronously record even small changes in the parameters directly in the growing cultures. There were significant differences in the amount and kinetics of sulfide production between *B. subtilis* and *E. coli*. These differences are thought to have been due to the lack of the tripeptide glutathione in *Bacillus*. The data obtained in this study, together with those we reported earlier for *E. coli*, indicate that the production of sulfide may be a universal reaction, induced under different types of stresses by different bacterial species, and one of the potential sources of sulfide in nature. The stress-induced accumulation of H_2_S by *Bacillus* may play the role of a signaling molecule in intercellular and inter-organismal communications and facilitate the adaptation of bacteria and plants to environmental conditions.

## Data Availability

The data used to support the findings of this study are available from the corresponding author upon reasonable request.

## Supplementary Materials

The following supporting information can be downloaded at https://www.mdpi.com/article/10.3390/microorganisms12091856/s1: Figure S1: Extracellular H_2_S accumulation in *B. subtilis* was determined using Pb(Ac)_2_ strips after incubation for 120 min under the tested conditions; Figure S2: Changes in intracellular (a) and extracellular (b) cysteine in *B. subtilis* under glucose starvation and treatment with chloramphenicol; Figure S3: The addition of glucose (10 mM) or NH_4_Cl (2 mM) to *B. subtilis* cultures deprived of glucose (a,c) or nitrogen (b,c) is accompanied by renewed oxygen consumption (a,b), a decrease in pH (a,b), and an increase in potential of the sulfide sensor by cessation of H_2_S production (c); and Figure S4: Extracellular H_2_S accumulation in *B. megaterium* was determined using Pb(Ac)_2_ after incubation for 120 min under the tested conditions.
