# Supplementary material for: Stress-Induced Sulfide Production by Bacillus subtilis and Bacillus megaterium
Source: Microorganisms. 2024 Sep 7;12(9):1856. doi: 10.3390/microorganisms12091856 (PMC11433681; doi:10.3390/microorganisms12091856)
Supplement: Supplementary file 1 [file microorganisms-12-01856-s001.zip › Figure S2.pdf]

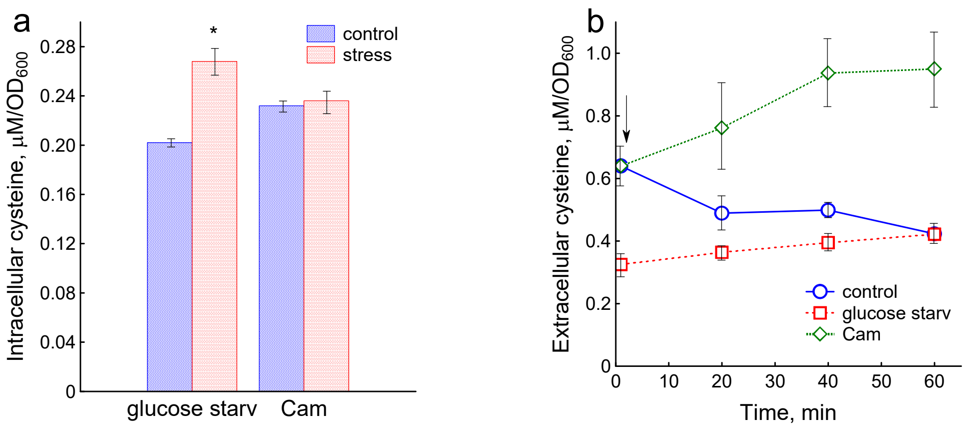

Figure S2. Changes in intracellular (a) and extracellular (b) cysteine in *B. subtilis* under glucose starvation and treatment with chloramphenicol.
