# Supplementary material for: Stress-Induced Sulfide Production by Bacillus subtilis and Bacillus megaterium
Source: Microorganisms. 2024 Sep 7;12(9):1856. doi: 10.3390/microorganisms12091856 (PMC11433681; doi:10.3390/microorganisms12091856)
Supplement: Supplementary file 1 [file microorganisms-12-01856-s001.zip › Figure S3.pdf]

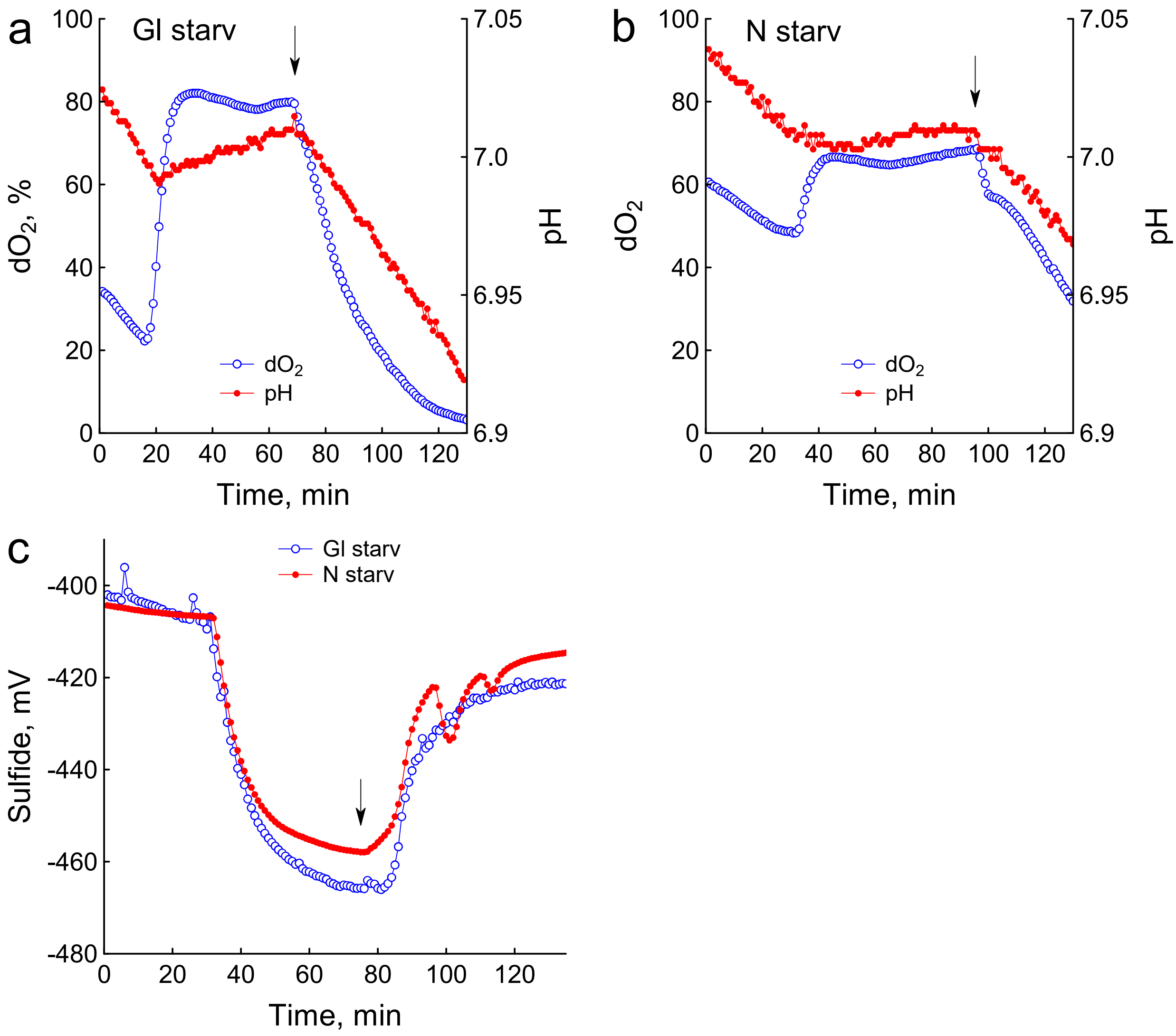

Figure S3. The addition of glucose (10 mM) or NH_4Cl (2 mM) to *B. subtilis* cultures deprived of glucose (a, c) or nitrogen (b, c) is accompanied by renewed oxygen consumption (a, b), a decrease in pH (a, b), and an increase in potential of the sulfide sensor by cessation of H_2S production (c). The time for adding substrates is indicated by arrows.
