# Supplementary figures and images for: Stress-Induced Sulfide Production by Bacillus subtilis and Bacillus megaterium
Source: Microorganisms. 2024 Sep 7;12(9):1856. doi: 10.3390/microorganisms12091856 (PMC11433681; doi:10.3390/microorganisms12091856)

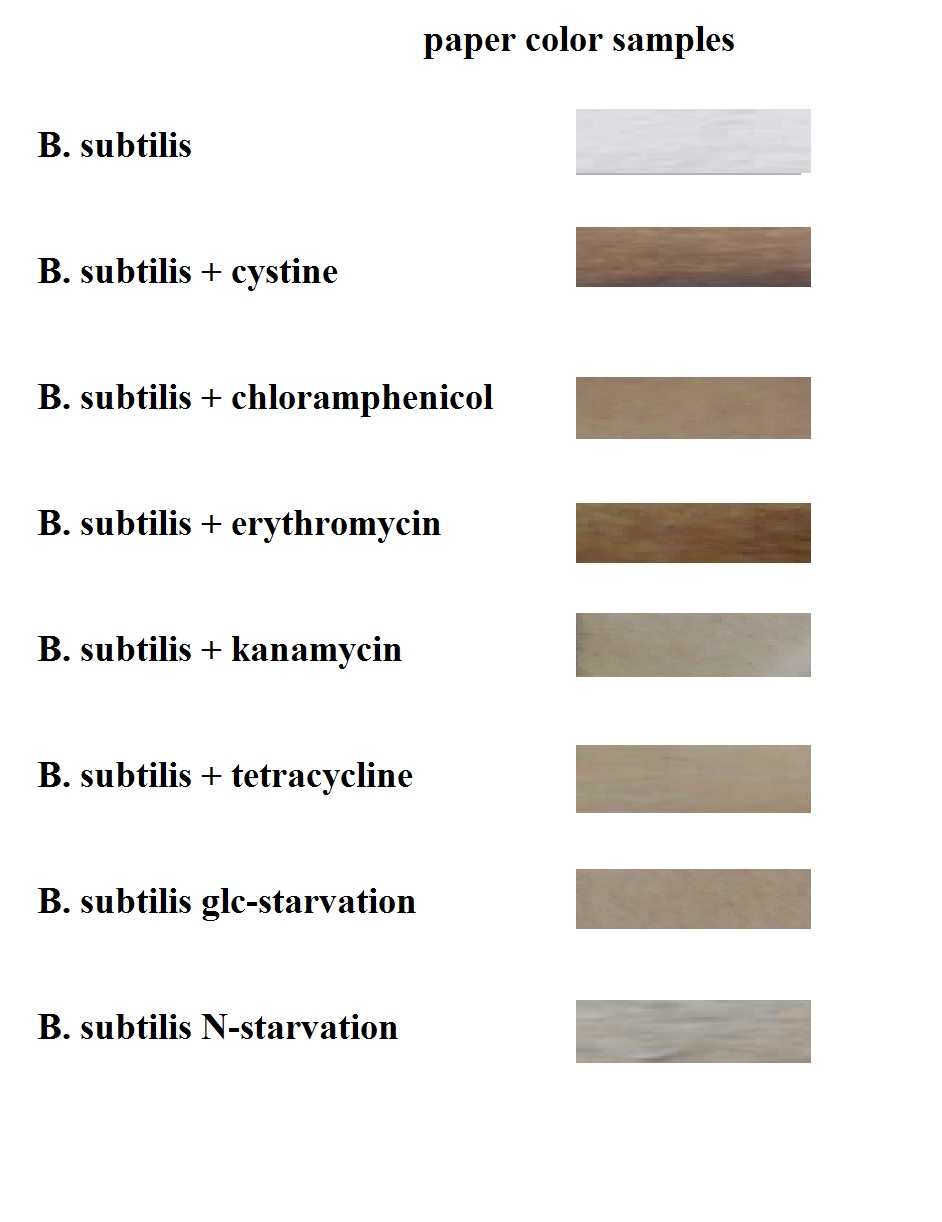

Supplement: Supplementary file 1 [file microorganisms-12-01856-s001.zip › Figure S1.jpg]

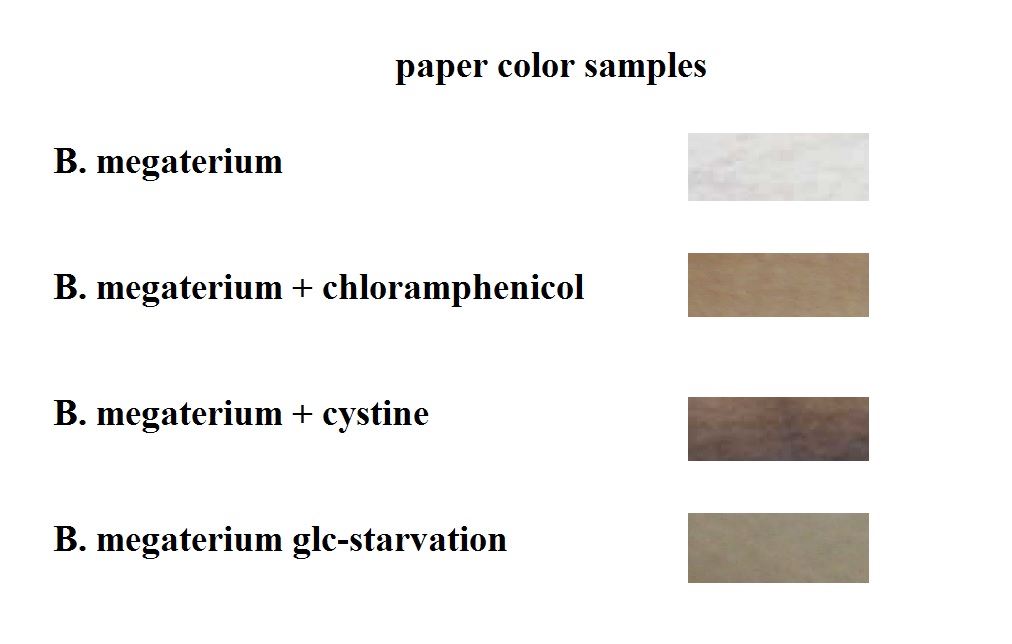

Supplement: Supplementary file 1 [file microorganisms-12-01856-s001.zip › Figure S4.jpg]
